# Circulating intercellular adhesion molecule 1 (ICAM-1), E-selectin and vascular cell adhesion molecule 1 (VCAM-1) in head and neck cancer

**DOI:** 10.1038/sj.bjc.6690057

**Published:** 1999-01

**Authors:** C-M Liu, T-S Sheen, J-Y Ko, C-T Shun

**Affiliations:** Department of Otolaryngology, College of Medicine, National Taiwan University, 7, Taipei, Taiwan Republic of China; Department of Pathology, College of Medicine, National Taiwan University, 7, Taipei, Taiwan Republic of China

**Keywords:** circulating adhesion molecule, intercellular adhesion molecule 1, E-selectin, vascular cell adhesion molecule 1, head and neck cancer

## Abstract

The sera from patients with nasopharyngeal carcinoma (*n* = 30), oral carcinoma (*n* = 22) and laryngeal carcinoma (*n* = 22) was extracted before treatment. The concentration of circulating intercellular adhesion molecule 1 (ICAM-1), E-selectin and vascular cell adhesion molecule 1 (VCAM-1) was measured by enzyme-linked immunoassay and compared with those from normal subjects (*n* = 20). The concentration of circulating ICAM-1, E-selectin and VCAM-1 was significantly increased in nasopharyngeal carcinoma. Correspondingly, VCAM-1 and E-selectin were significantly increased in laryngeal carcinoma, whereas only E-selectin was elevated in oral carcinoma. The concentrations of these adhesion molecules did not significantly differ with respect to the early and late stages of these carcinomas. Elevated levels of soluble adhesion molecules in the sera of cancer patients at three different head and neck regions, although appearing to be implicated in these tumour formations, may be unrelated to tumour progression. © 1999 Cancer Research Campaign


*British Journal of Cancer* (1999) **79**(2), 360–362
© 1999 Cancer Research Campaign

### Circulating intercellular adhesion molecule 1 (ICAM-1), E-selectin and vascular cell adhesion molecule 1 (VCAM-1) in head and neck cancer

**C-M Liu[1], T-S Sheen[1], J-Y Ko[1] and C-T Shun[2]**

Departments of [1]Otolaryngology and [2]Pathology, College of Medicine, National Taiwan University, 7, Chung-shan South Road, Taipei, Taiwan, Republic of China

**Summary** The sera from patients with nasopharyngeal carcinoma (*n* = 30), oral carcinoma (*n* = 22) and laryngeal carcinoma (*n* = 22) was extracted before treatment. The concentration of circulating intercellular adhesion molecule 1 (ICAM-1), E-selectin and vascular cell adhesion molecule 1 (VCAM-1) was measured by enzyme-linked immunoassay and compared with those from normal subjects (*n* = 20). The concentration of circulating ICAM-1, E-selectin and VCAM-1 was significantly increased in nasopharyngeal carcinoma. Correspondingly, VCAM-1 and E-selectin were significantly increased in laryngeal carcinoma, whereas only E-selectin was elevated in oral carcinoma. The concentrations of these adhesion molecules did not significantly differ with respect to the early and late stages of these carcinomas. Elevated levels of soluble adhesion molecules in the sera of cancer patients at three different head and neck regions, although appearing to be implicated in these tumour formations, may be unrelated to tumour progression.

**Keywords:** circulating adhesion molecule; intercellular adhesion molecule 1; E-selectin; vascular cell adhesion molecule 1; head and neck cancer

It is generally accepted that the direct interaction between adhesion molecules on the surfaces of inflammatory cells and vascular endothelium is thought to be necessary for cellular infiltration at the sites of inflammation (Roitt et al, 1993). In addition, ICAM-1 is normally present on endothelium and is known to be present in macrophages, fibroblasts, epithelial cells, lymphocytes and follicular dendritic reticulum cells (Rothlein et al, 1986; Koch et al, 1991). It is an inducible ligand for lymphocyte function-associated antigen 1 (LFA-1), and heavily influences cell–cell interactions in inflammatory and immune responses (Smith and Thomas, 1990). This interaction is also implicated in the various stages of tumour progression and metastasis (McCarthy et al, 1991). Circulating ICAM-1 in serum has been shown with elevated levels in several diseases (Rothlein et al, 1991; Seth et al, 1991), and higher levels associated with metastasis, tumour spread, and poor prognosis in gastrointestinal cancers (Tsujisaki et al, 1991), melanoma (Kageshita et al, 1992) and Hodgkin's disease (Pizzolo et al, 1993).

E-selectin, commonly known as endothelial leucocyte adhesion molecule-1 or ELAM-1, appears to be transiently endothelium specific in its expression, mediating neutrophil, monocyte and memory T-cell adhesion (Bevilacqua et al, 1993). VCAM-1 is also induced on endothelium, mediating adhesion of lymphocytes and monocytes; in addition, it is expressed in macrophages, follicular dendritic cells and neural cells (Rice et al, 1991; Birdsall et al, 1992). VCAM-1 and E-selectin may be involved in metastasis by mediating melanoma cells (Rice and Bevilacqua, 1989) and colon carcinoma cells (Lauri et al, 1991) to endothelium. From the study by Wenzel et al (1995), it suggests that the Sialyl Lewis$^x$ endothelial-selectin ligand interaction may be important in facilitating head and neck squamous cell carcinoma (HNSCC) cells to adhere during metastasis. As reported elsewhere, soluble forms of these molecules are associated with human malignancies (Banks et al, 1993).

In light of the above development, this study examines the concentration of circulating ICAM-1, E-selectin and VCAM-1 in nasopharyngeal carcinoma, oral carcinoma and laryngeal carcinoma and correlates their levels with disease status.

### MATERIALS AND METHODS

### Subjects

Patients with nasopharyngeal carcinoma (*n* = 30), oral carcinoma (*n* = 22) and laryngeal carcinoma (*n* = 22) were examined. Twenty

**Table 1** Clinical summary of control subjects and patients studied

| | Control subjects (*n* = 20) | NPC (*n* = 30) | OC (*n* = 22) | LC (*n* = 22) |
|---|---|---|---|---|
| Sex | | | | |
| Men | 8 | 23 | 17 | 21 |
| Women | 12 | 7 | 5 | 1 |
| Age (years) | | | | |
| Mean ± s.d. | 38.3 ± 4.0 | 48.61 ± 12.4 | 50.3 ± 9.5 | 64.6 ± 8.6 |
| Range | 19–63 | 19–69 | 32–64 | 44–81 |
| Stage I and II | | 13 | 14 | 12 |
| Stage III and IV | | 17 | 8 | 10 |

NPC, nasopharyngeal carcinoma; OC, oral carcinoma; LC, laryngeal carcinoma; s.d., standard deviation. Staging was performed according to AJCC system.

*Received 19 January 1998*
*Revised 6 May 1998*
*Accepted 13 May 1998*

*Correspondence to:* C-M Liu, Department of Otolaryngology, National Taiwan University Hospital, 7, Chung-Shan South Road, Taipei, Taiwan, ROC

**Table 2** Serum adhesion molecule levels in three head and neck cancers

| | Control subjects ($n$ = 20) | NPC ($n$ = 30) | OC ($n$ = 22) | LC ($n$ = 22) |
|---|---|---|---|---|
| ICAM-1 | 215.4 ± 10.2[a] | 269.9 ± 19.0* | 219.0 ± 19.2 | 157.5 ± 12.6 |
| E-selectin | 41.7 ± 4.0 | 71.3 ± 5.3* | 70.5 ± 12.1* | 64.8 ± 9.2* |
| VCAM-1 | 837.0 ± 39.8 | 1168.9 ± 92.8* | 985.0 ± 85.5 | 1155.2 ± 115.8* |

NPC, nasopharyngeal carcinoma; OC, oral carcinoma; LC, laryngeal carcinoma. [a]Mean ± standard error mean (ng $ml^{-1}$); *significantly higher than in control subjects $P < 0.05$ by Student's $t$-test.

**Table 3** Comparison of serum levels of adhesion molecules between early and late stages

| | ICAM-1 | E-selectin | VCAM-1 |
|---|---|---|---|
| NPC | | | |
| Stage I and II ($n$ = 13) | 252.5 ± 23.9[a] | 70.0 ± 8.3 | 1188.5 ± 155.7 |
| Stage III and IV ($n$ = 17) | 289.1 ± 29.2 | 72.3 ± 7.1 | 1154.0 ± 116.6 |
| OC | | | |
| Stage I and II ($n$ = 14) | 224.0 ± 19.5 | 65.1 ± 13.7 | 997.0 ± 93.7 |
| Stage III and IV ($n$ = 8) | 208.9 ± 44.9 | 90.7 ± 23.6 | 918.6 ± 183.7 |
| LC | | | |
| Stage I and II ($n$ = 10) | 140.8 ± 15.9 | 62.5 ± 13.8 | 1284.0 ± 188.1 |
| Stage III and IV ($n$ = 12) | 170.0 ± 18.2 | 66.8 ± 12.9 | 1132.5 ± 143.7 |

[a]Mean ± standard error mean (ng $ml^{-1}$); NPC, nasopharyngeal carcinoma; OC, oral carcinoma; LC, laryngeal carcinoma.

people who received laryngomicrosurgery for vocal polyps or nodules and without regional or systemic disorders were used as control subjects. Informed consent was obtained from all subjects. Table 1 summarizes the patients and control subjects studied herein, including gender, age and staging of carcinomatous status. The staging was according to the AJCC Staging System (1988).

### Blood samples

Blood samples were obtained before definite treatment was begun. Blood was centifuged at 1500 r.p.m. for 15 min, at room temperature. Finally, serum was separated and frozen until further use.

### Immunoassay

The concentration of sICAM-1, sE-selectin and sVCAM-1 in serum was determined by enzyme-linked immunoassay kits (ELISA) from R&D Systems (Minneapolis, MN, USA), and were used according to the manufacturer's instructions. The sensitivities were 7 ng $ml^{-1}$ for sICAM-1, 2 ng $ml^{-1}$ for sE-selectin and 100 ng $ml^{-1}$ for sVCAM-1.

### Statistical analysis

The statistical significance was evaluated with Student's $t$-test. A $P$-value of less than 0.05 was deemed statistically significant.

### RESULTS

According to Table 2, sICAM-1, sE-selectin and sVCAM-1 in the area of nasopharyngeal cancer, sE-selectin in oral cancer, and sE-selectin and sVCAM-1 in laryngeal cancer are significantly higher than those in control subjects, although the sICAM-1 in laryngeal cancer is significantly lower.

According to the staging systems, the status of these malignancies was divided into early (stage I and II) and late (stage III and IV) stages. sICAM-1, sE-selectin and sVCAM-1 do not differ in terms of concentrations between the early and the late stages of nasopharyngeal, oral and laryngeal carcinoma (Table 3).

### DISCUSSION

The cellular source and the mechanisms for releasing the soluble components of these endothelial adhesion molecules, although not well known, could involve either shedding or enzymatic cleavage from endothelial cells, leucocyte surfaces or tumour cells. Previous works have indicated that the cellular expression of ICAM-1 on normal and malignant epithelial tissue including melanoma cell lines (Natali et al, 1990; Becker et al, 1991; Giavazzi et al, 1992) and renal cell lines (Tomita et al, 1990) can be augmented by γ-interferon (IFN-γ), interleukin 1 (IL-1) and tumour necrosis factor (TNF-α) (Maio et al, 1989; Vanky et al, 1990; Azuma et al, 1992). Notably, TNF can cause the release of ICAM-1, VCAM-1 and E-selectin from endothelial cells of human umbilical vein (Pigott et al, 1992). IFN-γ can also induce expression and shedding of ICAM-1 from gastric cell lines (Harning et al, 1991). However, the clinical significance and the interaction between these molecules still remain relatively unknown. Yamamoto et al (1994) measured the circulating ICAM-1 in the sera of oral diseases, indicating that the circulating ICAM-1 was not elevated in the sera of oral squamous cell carcinoma patients. Similarly, in this series, the circulating ICAM-1 was not elevated in the sera of patients with oral cancer and laryngeal cancer in this series. However, circulating ICAM-1, E-selectin and VCAM-1 were elevated in the sera of patients with nasopharyngeal carcinoma (NPC), i.e. a common human epithelial carcinoma in South-East Asia. The discrepancy of the level of soluble ICAM-1 among these three groups of patients of head and neck carcinomas might be attributed to either the different immunological reaction profiles or a cell-specific response. According to previous investigators, soluble IL-2 receptor, IFN-γ and TNF-α were elevated in NPC patients (Hsu et al, 1991; Kuo et al, 1994). These factors might contribute towards the cellular expression and shedding of ICAM-1, E-selectin and VCAM-1 as shown in these NPC patients.

VCAM-1 and E-selectin are largely absent on the endothelium of normal tissue vessels (Kuzu et al, 1993). VCAM-1 is present on lymphoid dendritic cells, some tissue macrophages and renal parietal epithelium in addition to activated endothelial cells (Rice et al, 1991). VCAM-1 might be involved in metastasis of melanoma (Rice et al, 1989). Interestingly, elevated levels of sVCAM-1 were noted in several human malignancies such as in the ovary and breast, outside of the head and neck region (Banks et al, 1993). Expression of E-selectin is endothelium-specific (Bevilacqua and Nelson, 1993), and E-selectin-mediated adhesion of colon carcinoma and head and neck squamous cell carcinoma cells to endothelium is suggested to be associated with metastasis (Lauri et al, 1991; Wenzel et al, 1995). Soluble E-selectin is evaluated as a marker for endothelial damage after activation by cytokines (Gearing and Newman, 1993), the levels are higher in ovarian, breast and gastrointestinal cancers (Banks et al, 1993). The increased levels of sE-selectin in the patients sera of NPC, oral and laryngeal cancers in this series might be attributed to the shedding of endothelially bound E-selectin in the tumour tissues.

© Cancer Research Campaign 1999 

Thus, this E-selectin might be a potential marker for tumour invasiveness or angiogenesis in head and neck cancer.

The differences arising between the levels of sICAM-1, sVCAM-1 and sE-selectin in these three head and neck cancers might be attributed to the nature of the tumour, cytokines responsible for adhesion molecule expression, shedding regardless of whether they are tumour-derived or derived from surrounding host tissues, and kinetics of expression and shedding.

The progression of a tumour from benign delimited proliferation to invasive and metastasic growth depends on angiogenesis, enhanced extracellular matrix degradation via tumour- and host-secreted proteases, tumour cell migration and modulation of tumour cell adhesion. Each individual component is multifaceted (Price et al, 1997). The fact that sICAM-1, sVCAM-1 and sE-selectin did not significantly differ in terms of levels between early and late stages of these three head and neck cancers accounts for why these soluble adhesion molecules appear not to be the only factor in their tumour progression.

Immunologically, shedding of adhesion molecules by activated endothelial cells and tumour cells might not only block their counter ligands on immunocompetent cells, but also allow the tumour cells to escape from surveillance by cytotoxic T-cells and natural killer cells, thereby promoting metastasis. These shedding adhesion molecules might also prevent tumour cells from adhering to endothelial cells during extravasation. Future investigations on the function and interaction of these adhesion molecules may clarify the mechanism of tumour progression and metastasis.

### ACKNOWLEDGEMENT

The authors would like to thank Miss Hsiao for her technical assistance and Mr Ted Knoy for his assistance in correcting the English text.

### REFERENCES

Azuma A, Yagita H, Okumura K and Niitani H (1992) Induction of intercellular adhesion molecule 1 on small cell lung carcinoma cell lines by γ-interferon enhances spontaneous and bispecific anti-CD 3 × antitumor antibody-directed lymphokine-activated killer cell cytotoxicity. *Cancer Res* **52**: 4890–4894

Banks RE, Gearing AJH, Hemingway IK, Norfolk DR, Perren TJ and Selby PJ (1993) Circulating intercellular adhesion molecule-1 (ICAM-1), E-selectin and vascular cell adhesion molecule-1 (VCAM-1) in human malignancies. *Br J Cancer* **68**: 122–124

Becker JC, Dummer R, Hartmann AA, Berg G and Schmidt RE (1991) Shedding of ICAM-1 from human melanoma cell lines induced by IFNγ and tumor necrosis factor α: functional consequences on cell-mediated cytotoxicity. *J Immunol* **147**: 4398–4401

Bevilacqua MP and Nelson RM (1993) Selectins. *J Clin Invest* **91**: 379–387

Birdsall HH, Lane C, Ramser MN and Anderson DC (1992) Induction of VCAM-1 and ICAM-1 on human neural cells and mechanisms of mononuclear leukocyte adhesion. *J Immunol* **148**: 2717–2723

Gearing AJH and Newman W (1993) Circulating adhesion molecules in disease. *Immunol Today* **14**: 506–512

Giavazzi R, Chirivi RGS, Garofalo A, Rambaldi A, Hemingway IK, Pigott R and Gearing AJH (1992) Soluble intercellular adhesion molecule 1 is released by human melanoma cells and is associated with tumor growth in nude mice. *Cancer Res* **52**: 2628–2630

Harning R, Mainolfi E, Bystryn JC, Henn M, Merluzzi VJ and Rothlein R (1991) Serum levels of circulating intercellular adhesion molecule 1 in human malignant melanoma. *Cancer Res* **51**: 5003–5005

Hsu MM, Ko JY and Chang YL (1991) Elevated levels of soluble interleukine-2 receptor, and tumor necrosis factor in naspharygeal carcinoma. *Arch Otolaryngol Head Neck Surg* **117**: 1257–1259

Kageshita Y, Yoshii A, Kimura T and Ono T (1992) Analysis of expression and soluble form of intercellular adhesion molecule-1 in malignant melanoma. *J Dermatol* **19**: 836–840

Koch AE, Burrows JC, Haines GK, Calos TM, Harlan JM and Leibovich SJ (1991) Immunolocalization of endothelial and leukocyte adhesion molecules in human rheumatoid and osteoarthritic synovial tissues. *Lab Invest* **64**: 313–320

Kuo WR, Yu HS, Chang KL, Juan KH, Jan YS and Yu CL (1994) Increased production of tumor necrosis factor-α and release of soluble CD 4 and CD 8 molecules, but decreased responsiveness to phytohemagglutinin in patients with nasopharyngeal carcinoma. *J Formos Med Assoc* **93**: 569–575

Kuzu I, Bicknell R, Fletcher CD and Gatter KC (1993) Expression of adhesion molecules on the endothelium of normal tissue vessels and vascular tumors. *Lab Invest* **69**: 322–328

Lauri D, Needham LA, Martin-Padura I and Dejana E (1991) Tumor cell adhesion to endothelial cells: endothelial leukocyte adhesion molecule-1 as an inducible adhesive receptor specific for colon carcinoma cells. *J Natl Cancer Inst* **83**: 1321–1324

Maio M, Gulwani B, Langer JA, Kerbel RS, Duigou GJ, Fisher PB and Ferrone S (1989) Modulation by interferons of HLA antigen, high-molecular-weight melanoma-associated antigen, and intercellular adhesion molecule-1 expression by cultured melanoma cells with different metastatic potential. *Cancer Res* **49**: 2980–2987

McCarthy JB, Skubitz APN, Iida J, Mooradian DL, Wilke MS and Furcht LT (1991) Tumor cell adhesive mechanisms and their relationship to metastasis. *Semin Cancer Biol* **2**: 155–167

Natali P, Nicotra MR, Cavaliere E, Bigotti A, Romano G, Temponi M and Ferrone S (1990) Differential expression of intercellular adhesion molecule-1 in primary and metastatic melanoma lesion. *Cancer Res* **50**: 1271–1278

Pigott R, Dillon LP, Hemingway IH and Gearing AJH (1992) Soluble forms of E-selectin, ICAM-1 and VCAM-1 are present in the supernatants of cytokine activated endothelial cells. *Biochem Biophys Res Commun* **187**: 584–589

Pizzolo G, Vinante F, Nadali G, Ricetti MM, Morosato L, Marrocchella R and Vincenzi C (1993) ICAM-1 tissue over-expression associated with increased serum levels of its soluble form in Hodgkin's disease. *Br J Haematol* **84**: 161–162

Price JT, Bonovich MT and Kohn EC (1997) The biochemistry of cancer dissemination. *Crit Rev Biochem Mol Biol* **32**: 175–253

Rice GE and Bevilacqua MP (1989) An inducible endothelial cell surface glycoprotein mediates adhesion. *Science* **246**: 1303–1306

Rice GE, Munro JM, Corless C and Bevilacqua MP (1991) Vascular and nonvascular expression of INCAM-110. *Am J Pathol* **138**: 385–393

Roitt I, Brostoff J and Male D (1993) Cell migration and inflammation. In *Immunology* pp. 13.1–13.8. Mosby: London

Rothlein R, Dustin ML, Marlin SD and Springer TA (1986) A human intercellular adhesion molecule (ICAM-1) distinct from LFA-1. *J Immunol* **137**: 1270–1274

Rothlein R, Mainolf EA, Czajkowski M and Marlin SD (1991) A form of circulating ICAM-1 in human serum. *J Immunol* **147**: 3788–3793

Seth R, Raymond FD and Mukgoba MW (1991) Circulating ICAM-1 isoforms: diagnostic prospects for inflammatory and immune disorders. *Lancet* **338**: 83–84

Smith MEF, ThoSmith MEF and Thomas JA (1990) Cellular expression of lymphocyte function associated antigens and the intercellular adhesion molecule-1 in normal tissue. *J Clin Pathol* **43**: 898–900

Tomita Y, Nishiyama T, Watanabe H, Fujiwara M and Sato S (1990) Expression of intercellular adhesion molecule-1 (ICAM-1) on renal cell cancer: possible significance in host immune response. *Int J Cancer* **46**: 1002–1006

Tsuhsaki M, Imai K, Hirata H, Hanzawa Y, Masuya J, Nakano T and Sugiyama T (1991) Detection of circulating intercellular adhesion molecule-1 antigen in malignant diseases. *Clin Exp Immunol* **85**: 3–8

Vanky F, Wang P, Patarroyo M and Klein E (1990) Expression of the adhesion molecule ICAM-1 and major histocompatibility complex class I antigens on human tumor cells is required for their interaction with autologous lymphocytes in vitro. *Cancer Immunol Immunother* **31**: 19–27

Wenzel CT, Scher RL and Richtsmeier WJ (1995) Adhesion of head and neck squamous cell carcinoma to endothelial cells. *Arch Otolaryngol Head Neck Surg* **121**: 1279–1286

Yamanoto T, Yoneda K, Ueta E and Osaki T (1994) Serum cytokines, interleukin-2 receptor, and soluble intercellular adhesion molecule-1 in oral disorders. *Oral Surg Oral Med Oral Pathol* **78**: 727–735

 © Cancer Research Campaign 1999
